# Diet-induced obesity impairs salt-dependent downregulation of NCC activity

**DOI:** 10.1038/s41440-026-02798-z

**Published:** 2026-09-11

**Authors:** Chiharu Kondo, Kohei Ueda, Tatsuo Shimosawa, Kaoruko Iida

**Affiliations:** 1 Department of Food and Nutritional Science, Graduate School of Humanities and Sciences, Ochanomizu University, Bunkyo, Tokyo Japan; 2 Department of Physiology, School of Medicine, International University of Health and Welfare, Narita, Chiba Japan; 3 Department of Clinical Laboratory, Graduate School of Medicine, International University of Health and Welfare, Narita, Chiba Japan; 4 The Institute for Human Life Sciences, Ochanomizu University, Bunkyo, Tokyo Japan

**Keywords:** Na-Cl cotransporter (NCC), salt sensitivity, renal sodium handling, diet-induced obesity (DIO)

## Abstract

Obesity is associated with increased salt sensitivity and hypertension, but the mechanisms underlying altered renal sodium handling remain unclear. The Na-Cl cotransporter (NCC) is a key regulator of sodium reabsorption in the distal convoluted tubule and is dynamically regulated in response to changes in electrolyte balance. This study investigated whether diet-induced obesity (DIO) alters NCC responsiveness to salt loading in vivo and explored the underlying mechanisms using an ex vivo kidney slice model. Male C57BL/6 J mice were fed either a control or a high-fat (HF) diet for 22 weeks, followed by 2 weeks of NaCl loading. HF-fed mice developed obesity, hyperglycemia, hyperinsulinemia, and elevated systolic blood pressure. Although NaCl loading increased urinary sodium excretion and decreased serum aldosterone in both control and obese mice, the increase in urinary sodium excretion was attenuated in obese mice, suggesting enhanced renal sodium reabsorption. In addition, NaCl loading reduced NCC phosphorylation in control mice, whereas this response was attenuated in DIO mice, with no change in total NCC expression. Notably, STE20/SPS1-related proline-alanine-rich protein kinase (SPAK) phosphorylation was not significantly different among groups. In ex vivo kidney slice experiments, high-concentration insulin attenuated the high-potassium-induced suppression of NCC phosphorylation, whereas high glucose had no effect. These findings suggest that DIO impairs the normal suppression of NCC activity in response to salt loading without affecting basal NCC expression. Ex vivo findings suggest that insulin may modulate NCC responsiveness, although whether this mechanism contributes to NCC regulation in vivo remains uncertain. Impaired NCC responsiveness may promote renal sodium retention and could represent one potential mechanism linking obesity to altered sodium handling and blood pressure regulation.

**Figure:**
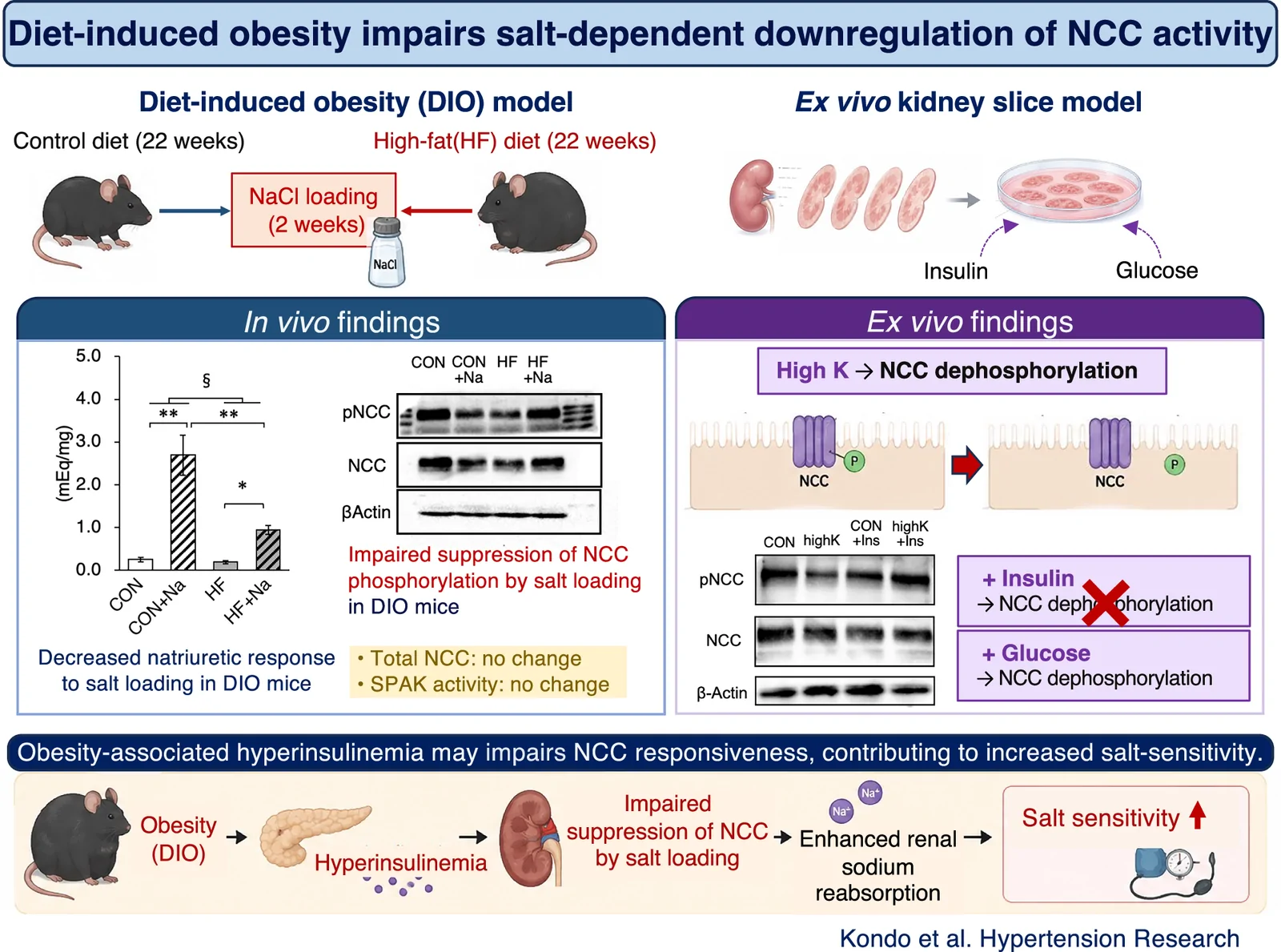

## Introduction

Hypertension is a major risk factor for cardiovascular disease, the leading cause of mortality worldwide. One of the key concepts in understanding the pathophysiology of hypertension is salt sensitivity, which refers to interindividual differences in blood pressure responses to changes in dietary salt intake. Physiological mechanisms tightly regulate sodium (Na) balance in response to changes in salt intake. However, in salt-sensitive individuals, the excretion of dietary salt is reduced, resulting in increased blood pressure following salt intake. Hypertension in response to salt intake is referred to as salt-sensitive hypertension and is associated with a poorer prognosis than salt-resistant hypertension [1]. Classical epidemiological studies have suggested that cardiovascular events and mortality are higher in salt-sensitive individuals than in salt-resistant individuals [2, 3].

Salt sensitivity is influenced by multiple factors, including genetic background, sex, and body composition [4, 5]. Among these, obesity has emerged as an important contributor to salt-sensitive hypertension. Obesity is thought to promote renal sodium reabsorption through dysregulation of neural and endocrine systems, such as hyperactivation of the sympathetic nervous system and the renin-angiotensin-aldosterone system, thereby contributing to enhanced salt sensitivity and hypertension [6, 7]. Indeed, clinical studies have demonstrated that obese patients with metabolic syndrome exhibit increased salt sensitivity [8, 9]. However, the molecular mechanisms by which obesity alters renal sodium reabsorption remain incompletely understood.

The Na-Cl cotransporter (NCC) is a sodium transporter expressed in the distal convoluted tubule of the kidney. Although NCC accounts for only approximately 5–10% of total sodium reabsorption in the renal tubules, alterations in its activity can significantly affect whole-body sodium balance [10]. NCC is activated through phosphorylation by its principal upstream kinase, STE20/SPS1-related proline/alanine-rich kinase (SPAK), thereby promoting sodium reabsorption [10, 11]. NCC activity is dynamically modulated in response to dietary sodium intake. Under low-sodium conditions, NCC is activated via SPAK-mediated phosphorylation to facilitate sodium retention, whereas under high-sodium conditions, NCC activity is suppressed in association with decreased SPAK activity [12]. Thus, dysregulation of NCC activity may contribute directly to the development of salt-sensitive hypertension.

Given its important role in sodium reabsorption, NCC has attracted considerable attention in the context of obesity-associated changes in salt sensitivity. However, most experimental studies investigating the relationship between obesity and NCC have used genetic models of obesity, such as Zucker obese rats [13–15] or db/db mice [16, 17], which may not fully recapitulate the pathophysiology of lifestyle-related human obesity. Moreover, although these studies suggest that obesity enhances basal NCC expression and/or activity, its impact on the dynamic response of NCC to salt loading remains insufficiently characterized. In contrast, studies using diet-induced obesity (DIO) models are limited [18, 19], and their findings have been inconsistent with those obtained from genetic obesity models. Regarding the underlying mechanisms, hyperinsulinemia and hyperglycemia have been proposed as potential contributors to NCC activation; however, much of the supporting evidence has come from in vitro studies [15, 20, 21] and may therefore not adequately reflect the complexity of in vivo renal physiology.

In this study, we established a DIO mouse model and subjected the mice to NaCl loading to investigate the role of NCC in obesity-associated alterations in salt sensitivity. Furthermore, to explore the underlying mechanisms, we assessed the effects of glucose and insulin on the dynamic regulation of NCC using an ex vivo kidney slice model.

## Materials and methods

### Animal experiments

Male C57BL/6 J mice (9 weeks old, *n* = 26; Sankyo Labo Service, Tokyo, Japan) were housed in a temperature-controlled room (22 ± 2 °C) under a 12-h light/dark cycle with free access to food and water. After a one-week acclimatization period, the mice were randomly assigned to four groups: control (CON), CON+Na, high fat (HF), and HF+Na. Mice in the CON and CON+Na groups were fed a standard diet based on AIN-93M, whereas those in the HF and HF+Na groups were fed a high-fat diet containing 60% of energy from fat for 22 weeks. The ingredients of the diet were purchased from Oriental Yeast Co. (Tokyo, Japan), and the composition of each diet is shown in Supplementary Table 1. After 22 weeks, NaCl loading was introduced for 2 weeks. The diets were replaced with either a standard diet supplemented with 4% NaCl (w/w) for the CON+Na group or a high-fat diet supplemented with 4% NaCl (w/w) for the HF+Na group, and feeding continued for a further 2 weeks, resulting in a total experimental period of 24 weeks.

The body weight of the mice was measured weekly throughout the experimental period. At the end of the experiment, the mice were fasted for 4 h and anesthetized with isoflurane to collect blood samples. The mice were then euthanized by cervical dislocation under anesthesia, and the kidneys were immediately excised. All animal procedures were approved by the Animal Ethics Committee of Ochanomizu University (Approval No. 25012) and were conducted according to the Guidelines for proper conduct of animal experiments by the Science Council of Japan.

### Biochemical analysis of blood and urine samples

The blood samples were centrifuged at 8000 rpm for 15 min in a microcentrifuge (Model 3500, Kubota, Tokyo, Japan), and the supernatant was transferred to a new tube and stored at −80 °C until analysis. Blood glucose, triglyceride, non-esterified fatty acid (NEFA), and blood urea nitrogen (BUN) levels were measured using the biochemical colorimetric assay kits LabAssay™ Glucose (FUJIFILM Wako Pure Chemical Corporation, Osaka, Japan), LabAssay™ Triglyceride (FUJIFILM Wako Pure Chemical Corporation), LabAssay™ NEFA (FUJIFILM Wako Pure Chemical Corporation), and Wako L-type UN reagent (FUJIFILM Wako Pure Chemical Corporation) according to the manufacturers’ protocols. All absorbance measurements were performed using an EnSpire® microplate reader (PerkinElmer, Waltham, MA, USA). Plasma insulin and aldosterone concentrations were evaluated using a mouse insulin ELISA kit (MS300; Takara Bio, Shiga, Japan) and an aldosterone ELISA kit (ADI-901-173; Enzo Life Sciences, NY, USA), respectively, according to the manufacturers’ instructions. Absorbance was measured using a Multiskan FC microplate reader (Thermo Fisher Scientific JP, Tokyo, Japan). HOMA-R was calculated using the following formula: blood glucose (mg/dL) × serum insulin (ng/mL) × 26 (μU/ng) / 405.

Urine samples were collected by spontaneous urination or gentle bladder massage after blood pressure measurement and stored at −80 °C until analysis. Urinary sodium, potassium, and creatinine concentrations were measured at SRL Inc. (Tokyo, Japan) in samples where an adequate volume was successfully collected.

### Blood pressure measurement

Systolic blood pressure was measured noninvasively using a tail-cuff device (BP-98A-L, Softron, Tokyo, Japan) 1–2 days before the end of the experiment. Seven consecutive measurements were taken, and the average of five values, excluding the highest and lowest values, was calculated for each mouse.

### Ex vivo kidney slice experiment

Ex vivo experiments were performed following a method reported previously [22], with slight modifications. Age-matched male mice (3–4 months old) were used for the preparation of kidney slices. Both kidneys were quickly removed from the mice under isoflurane anesthesia and sectioned into 300 μm-thick slices using a vibratome (VT1000S, Leica Microsystems, Wetzlar, Germany). Slices were placed on 0.4-μm pore inserts in 6-well plates (Greiner Bio-One, Kremsmünster, Austria) and equilibrated in Krebs-type buffer (98.5 mM NaCl, 35 mM NaHCO₃, 3 mM KCl, 1 mM NaH₂PO₄, 2.5 mM CaCl₂, 1.8 mM MgCl₂, and 25 mM glucose) at 30.5 °C under 5% CO₂ for 10 min. They were then incubated for the indicated times in the following buffers: control (CON; 3 mM K⁺), or high potassium (high K; 10 mM K⁺) buffer. In some experiments, buffers supplemented with insulin (500 nM), or additional glucose (25 mM) were used. After incubation, the slices were rapidly frozen in liquid nitrogen and used for Western blot analysis.

### Quantitative Real-time PCR

Total RNA was extracted from snap-frozen kidney tissue using RNAiso Plus reagent (Takara Bio) according to the manufacturer’s protocol. First-strand cDNA was synthesized from 1.0 μg of total RNA using ReverTra Ace™ qPCR RT Master Mix (Toyobo, Osaka, Japan). The diluted cDNA was used as a template to quantify relative mRNA levels. Quantitative real-time PCR reactions were prepared using SYBR^®^ Premix Ex Taq™ (Takara Bio) and performed on a StepOne Plus Real-Time PCR System (Thermo Fisher Scientific JP). The thermal cycling protocol was as follows: initial denaturation at 95 °C for 1 min, followed by 40 cycles of denaturation at 95 °C for 10 s and annealing/extension at 65 °C for 50 s. The gene expression levels of *Ncc* and *Renin* were normalized to those of glyceraldehyde-3-phosphate dehydrogenase (*Gapdh*). Primer sequences used for amplification are shown in Supplementary Table 2.

### Western blotting analysis

Animal tissue samples were homogenized in lysis buffer (50 mM Tris-HCl, 1 mM EDTA, 1 mM EGTA, 1 mM Na_3_VO4, 50 mM NaF, 1 mM DTT) containing 300 mM sucrose, 1× protease inhibitor cocktail (cOmplete^TM^, Sigma-Aldrich, St. Louis, MO, USA) and 1× phosphatase inhibitor (PhosSTOP, Sigma-Aldrich) according to a previous report [23]. The homogenates were centrifuged at 6000 rpm for 15 min at 4 °C, and the supernatants were collected for analysis. Ex vivo slice samples were homogenized in lysis buffer containing 1× protease inhibitor cocktail and 1× phosphatase inhibitor (Sigma-Aldrich), followed by centrifugation at 14,000 rpm for 15 min at 4 °C. The supernatants were used for subsequent analyses.

Equal amounts (8–10 μg per lane) of total protein were separated onto a 7% SDS–polyacrylamide gel and transferred onto an Immobilon-P PVDF membrane (Millipore, Billerica, MA, USA). Membranes were blocked with Blocking One-P (Nacalai Tesque, Kyoto, Japan) and incubated overnight at 4 °C with primary antibodies against NCC (1:15,000, AB3553, Millipore), phospho-NCC (1:4,000, S908B, University of Dundee, UK), SPAK (1:1,000, S669D, University of Dundee, UK), phospho-SPAK (1:2,000, 07-2273, Sigma-Aldrich) or β-actin (1:2,000, sc-47778, Santa Cruz Biotechnology, Dallas, TX, USA). After incubation, membranes were incubated for 1 h at room temperature (20–25 °C) with the appropriate secondary antibodies: anti-rabbit IgG (1:6,000, #7074, Cell Signaling Technology, Danvers, MA, USA) for NCC and phospho-SPAK, anti-sheep IgG (1:10,000, HAF016, R&D Systems, Minneapolis, MN, USA) for phospho-NCC and SPAK, or anti-mouse IgG (1:7,000, #7076, Cell Signaling Technology) for β-actin. Immunoreactive bands were detected using ECL™ Prime Western Blotting System (Cytiva, Marlborough, MA, USA) and visualized with the iBright Imaging System (Thermo Fisher Scientific JP). The optical density of each band was quantified using ImageJ software (National Institutes of Health, Bethesda, MD, USA).

### Statistical analyses

Data were presented as the mean ± standard error of the mean (SEM). All statistical analyses were performed using IBM SPSS Statistics for Windows (version 29; IBM, Tokyo, Japan). Normality of the variables was assessed using the Shapiro–Wilk test. For normally distributed data, two-way analysis of variance (ANOVA) was performed, followed by post hoc multiple comparisons. Tukey’s test was applied when variances were equal, whereas the Games–Howell test was used when variances were unequal. For non-normally distributed data, the Kruskal–Wallis test was used. A *p* value < 0.05 was considered statistically significant.

## Results

### DIO mice exhibit hyperglycemia and hyperinsulinemia

The initial and final body weights and the blood parameters at the end of the experiment in each group are shown in Table 1. Mice in the HF and HF+Na groups showed a significant increase in final body weight compared with the control groups (CON and CON+Na groups). Blood glucose and plasma insulin concentrations were also higher in the HF and HF+Na groups than in the control groups, resulting in significantly higher HOMA-R values, an index of insulin resistance. The unchanged plasma triglyceride and NEFA levels in HF-fed mice are consistent with previous reports showing that DIO mice develop obesity and insulin resistance without alterations in circulating lipid levels [24, 25]. There were also no significant differences in plasma BUN levels among the groups. NaCl supplementation had no significant effects on body weight or any of these blood parameters. In contrast, plasma aldosterone levels were significantly suppressed by NaCl supplementation in both control- and HF-fed mice.

**Table 1:** The body weight and blood parameters of mice

	CON	CON + Na	HF	HF + Na
**Initial body weight (g)**	24.1 ± 0.4	24.1 ± 0.2	23.6 ± 0.5	24.0 ± 0.5
**Final body weight (g)**	40.6 ± 1.2	40.0 ± 1.3	54.5 ± 1.5^**, ††^	53.5 ± 0.65^**, ††^
**Glucose (mg/dl)**	236.8 ± 11.0	228.8 ± 15.3	294.8 ± 15.6^*, †^	300.8 ± 16.7^*, †^
**Insulin (ng/ml)**	1.04 ± 0.15	1.05 ± 0.09	3.19 ± 0.28^**, ††^	2.60 ± 0.28^**, †^
**HOMA-R**	16.6 ± 3.4	15.7 ± 2.1	60.7 ± 7.1***, ^†††^	56.5 ± 7.6**, ^†††^
**Triglyceride (mg/ml)**	52.5 ± 3.7	54.7 ± 2.7	48.6 ± 7.0	41.8 ± 2.0
**NEFA (mg/dl)**	0.83 ± 0.07	0.98 ± 0.12	0.70 ± 0.05	0.78 ± 0.07
**BUN (mg/dl)**	20.0 ± 2.0	22.1 ± 1.6	22.4 ± 1.5	26.6 ± 1.3
**Aldosterone (pg/ml)**	184.8 ± 22.0	40.7 ± 6.7***	214.2 ± 22.1	55.0 ± 5.3^§§§^

Values are means ± SE. *N* = 5–7 in each group. *p < 0.05, **p < 0.01, ***p < 0.001 vs CON; ^†^*p* < 0.05, ^††^*p* < 0.01, ^†††^*p* < 0.001 vs CON + Na; ^§§§^*p* < 0.001 vs HF

*NEFA* non-esterified fatty acid, *BUN* blood urea nitrogen

### DIO mice exhibit elevated systolic blood pressure and decreased urinary sodium excretion

**Fig. 1:**
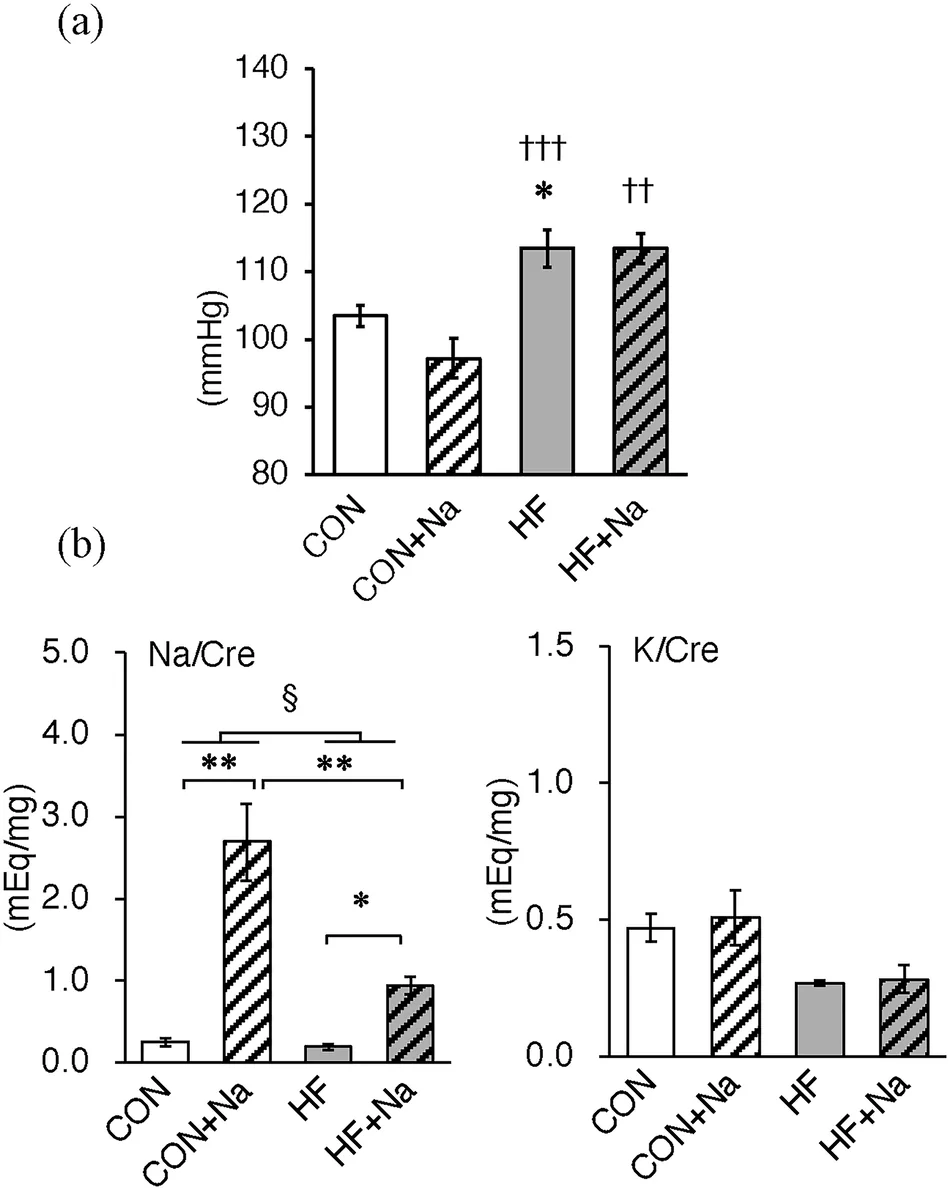
Systolic blood pressure and urinary sodium excretion in DIO mice. **a** Systolic blood pressure was measured 1–2 days before the end of the experiment in CON, CON+Na, HF, and HF+Na groups (*n* = 5–7 per group). Data are expressed as mean ± SEM. *p < 0.05 vs CON; ††p < 0.01, †††*p* < 0.001 vs CON+Na. **b** Urine samples were collected 1–2 days before the end of experiment in CON, CON+Na, HF, and HF+Na groups (*n* = 3–5 per group). Urinary sodium concentration was normalized to urinary creatinine levels. Data are expressed as mean ± SEM. *p < 0.05, **p < 0.01 between groups; ^§^*p* < 0.05 in interaction

Systolic blood pressure was significantly higher in the HF and HF+Na groups than in their respective control groups (CON and CON+Na groups) (Fig. 1a), indicating the development of obesity-induced hypertension. However, no additional increase in systolic blood pressure was observed in the HF+Na group compared with the HF group.

Urinary sodium concentration normalized to urinary creatinine was significantly higher in the CON+Na and HF+Na groups than in their respective control groups (CON and HF groups), indicating the effectiveness of NaCl loading. Furthermore, two-way ANOVA revealed a significant interaction between diet and NaCl loading (Fig. 1b), with reduced urinary sodium excretion in the HF+Na group. This suggests that urinary sodium reabsorption is increased in mice fed an HF diet. In contrast, urinary potassium excretion was not affected by either diet or NaCl loading.

### NaCl–induced suppression of NCC phosphorylation is attenuated in DIO mice

**Fig. 2:**
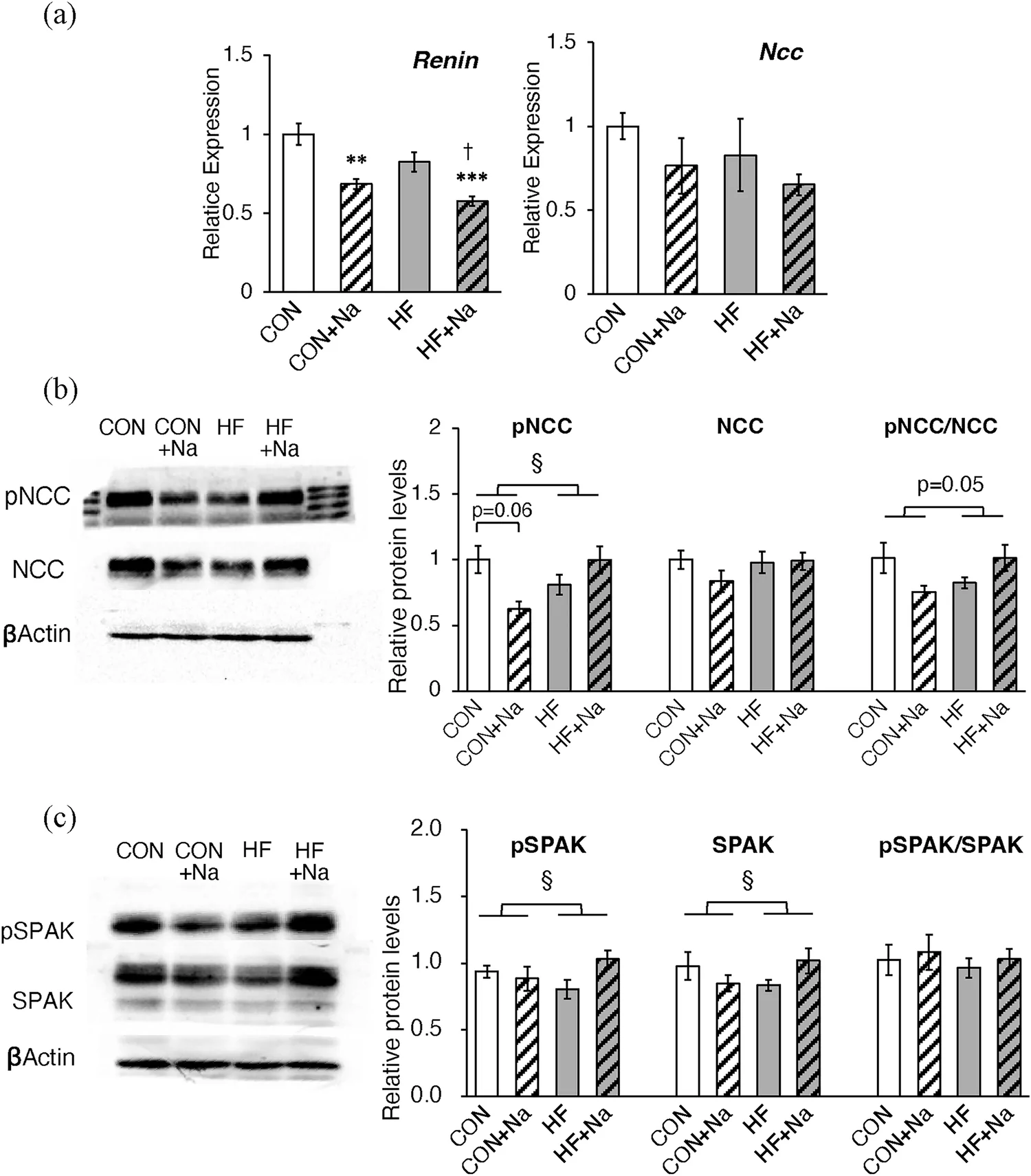
Impaired suppression of NCC phosphorylation by NaCl loading in DIO mice. **a** Relative mRNA expression levels of *Renin* and *Ncc*. Phosphorylated NCC (pNCC), total NCC, and pNCC/NCC ratios (**b**), and phosphorylated SPAK (pSPAK), total SPAK, and pSPAK/SPAK ratios (**c**) were quantified. Representative Western blot images are shown on the left. β-actin was used as a loading control. *n* = 5–7 per group. Data are expressed as mean ± SEM. **p < 0.01, ***p < 0.001 vs CON; ^†^*p* < 0.05 vs CON+Na; ^§^*p* < 0.05 in interaction

Gene expression of renin and NCC was quantified by RT-qPCR. *Renin* mRNA expression, which is known to be suppressed in response to high sodium intake, was significantly reduced in NaCl-loaded mice relative to their respective controls. However, no significant differences in *Ncc* expression levels were observed among the groups (Fig. 2a).

As NCC activity is regulated by phosphorylation, we measured phosphorylated NCC (pNCC) levels and the pNCC/NCC ratio by Western blotting (Fig. 2b). A significant interaction between diet and NaCl loading was observed in pNCC levels, with a decreasing trend in NCC phosphorylation in response to NaCl in mice fed a regular diet. A similar borderline trend was also observed in the pNCC/NCC ratio (p = 0.05). NCC protein levels did not differ among the groups, consistent with Ncc gene expression levels.

We also evaluated SPAK activity, as assessed by SPAK phosphorylation, which plays a pivotal role in NCC phosphorylation (Fig. 2c). A significant interaction was observed between diet and NaCl loading in the protein expression of both phosphorylated and total SPAK; however, no difference was observed in the pSPAK/SPAK ratio, suggesting that DIO does not affect SPAK activity.

### High insulin concentration alters NCC phosphorylation in kidney slices

**Fig. 3:**
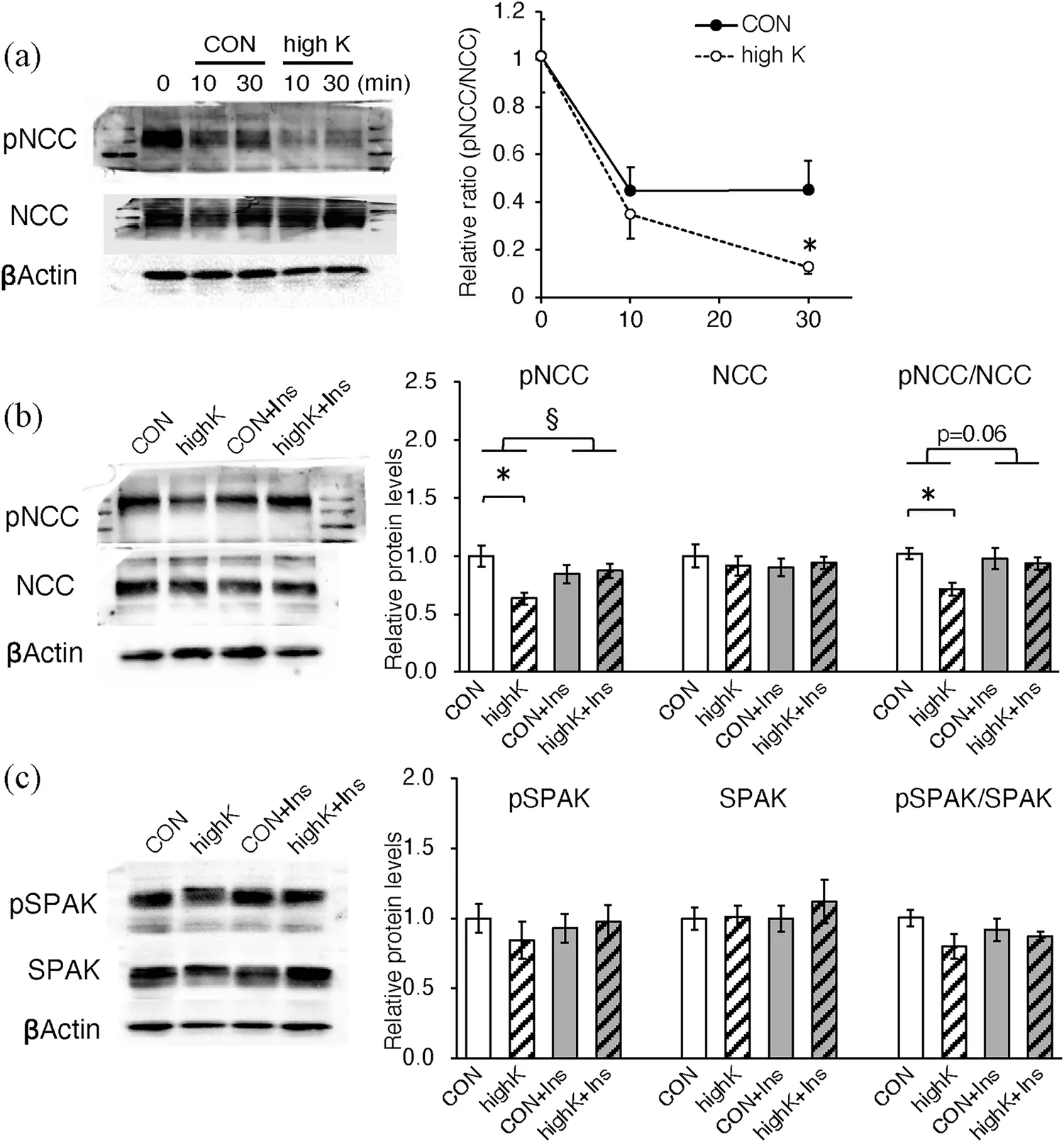
Effects of insulin on potassium-induced suppression of NCC phosphorylation in ex vivo kidney slices. **a** Representative Western blot images and a time course showing the time-dependent changes in the pNCC/NCC ratio (*n* = 4 slices per group). Representative Western blot images and quantification of pNCC, total NCC, and pNCC/NCC ratios (**b**), or of pSPAK, total SPAK, and pSPAK/SPAK ratios (**c**) (*n* = 9 slices per group). β-actin was used as a loading control. The conditions for each group are as follows: control (CON; 3 mM K⁺), high potassium (high K; 10 mM K⁺), CON with insulin (CON+Ins; 3 mM K⁺ and 500 nM insulin), and high K with insulin (high K+Ins; 10 mM K⁺ and 500 nM insulin). Data are expressed as mean ± SEM. *p < 0.05. ^§^*p* < 0.05 in interaction

After kidney slices were incubated in either control (3 mM K⁺) or high-K (10 mM K⁺) buffers for 0–30 min, NCC phosphorylation was significantly suppressed after 30 min incubation in the high-K buffer compared with that in the control group (Fig. 3a). Therefore, in subsequent experiments, the incubation time was set to 30 min to investigate the effects of insulin and glucose on NCC phosphorylation. When kidney slices were incubated in buffers containing a high concentration of insulin, the potassium-induced decrease in pNCC levels was significantly attenuated, as determined by two-way ANOVA, which revealed a significant interaction between insulin treatment and potassium concentration. A similar trend was observed for the pNCC/NCC ratio. The pNCC/NCC ratio was significantly reduced under high potassium conditions, but this reduction was abolished by insulin treatment (Fig. 3b). However, the protein expression levels of pSPAK and total SPAK were not significantly different among the groups, resulting in no significant difference in the pSPAK/SPAK ratio (Fig. 3c).

**Fig. 4:**
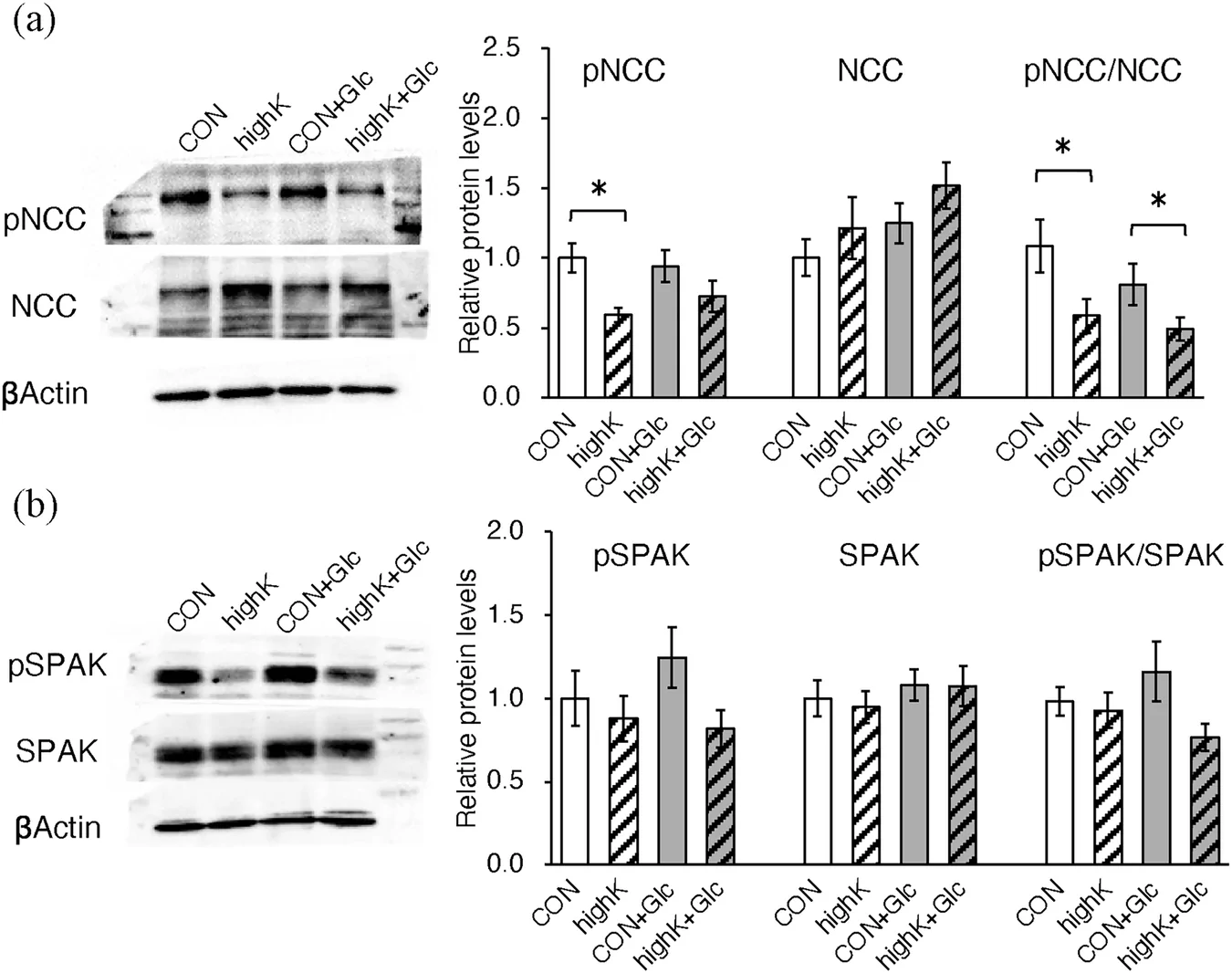
Effects of glucose on potassium-induced suppression of NCC phosphorylation in ex vivo kidney slices. Representative Western blot images and quantification of pNCC, total NCC, and pNCC/NCC ratios (**a**), or of pSPAK, total SPAK, and pSPAK/SPAK ratios (**b**) (*n* = 5 slices per group). β-actin was used as a loading control. The conditions for each group are as follows: control (CON; 3 mM K⁺), high potassium (high K; 10 mM K⁺), CON with glucose (CON+Glc; 3 mM K⁺ and 50 mM glucose), and high K with glucose (high K+Glc; 10 mM K⁺ and 50 mM glucose). Data are expressed as mean ± SEM. *p < 0.05

In contrast, the addition of a high concentration of glucose to the buffer had no significant effect on the reduction in pNCC abundance or the pNCC/NCC ratio induced by high potassium (Fig. 4a). Although the pNCC/NCC ratio showed a modest downward trend in glucose-treated groups, two-way ANOVA showed no significant main effect of glucose supplementation on NCC phosphorylation (*p* = 0.3). The protein expression levels of pSPAK and total SPAK were not significantly different among the groups, resulting in no significant difference in the pSPAK/SPAK ratio (Fig. 4b).

## Discussion

In this study, we investigated alterations in NCC activity in obesity using DIO mice. Our findings indicate that DIO impairs the normal suppression of NCC activity in response to NaCl loading. Many previous studies have investigated obesity-induced alterations in NCC using genetic obesity models, such as db/db mice and Zucker obese rats. These studies demonstrated increased NCC expression levels [13, 26], increased pNCC levels and/or pNCC/NCC ratios [15, 16], and enhanced NCC activity as assessed by the diuretic response to thiazide treatment [27], suggesting that obesity increases basal NCC activity in these models. In contrast, relatively few studies have examined NCC regulation in DIO models [18, 19]. These studies reported that mice fed a HF diet developed obesity and hypertension, whereas NCC activity remained unchanged. Consistent with previous findings, the present study showed that obesity alone did not enhance basal NCC activity; however, NaCl-induced suppression of NCC activity was attenuated in HF-induced obese mice. Taken together, these findings suggest that in DIO models that more closely reflect human obesity, obesity itself may not directly activate NCC. Rather, obesity may impair the normal responsiveness of NCC to NaCl loading.

To identify the factors contributing to the altered NCC response to NaCl loading in DIO mice, we conducted ex vivo experiments using kidney slices. Previous studies have suggested that high sodium intake indirectly modulates NCC activation in vivo, presumably via suppression of aldosterone-dependent effects, including changes in potassium homeostasis, whereas potassium loading directly inhibits NCC activation [28, 29]. Therefore, potassium loading was employed to suppress NCC activation in ex vivo experiments, to mimic the physiological conditions observed in the CON+Na group in vivo. As demonstrated in a previous study [22], potassium loading significantly suppressed NCC activity in ex vivo kidney slices. Under these conditions, insulin treatment attenuated the high potassium-induced decrease in pNCC levels, whereas high glucose treatment did not produce comparable effects. Although glucose treatment appeared to decrease the pNCC/NCC ratio, no significant main effect of glucose treatment was detected. Therefore, elevated glucose is unlikely to be a major contributor to the altered regulation of NCC phosphorylation observed in obesity.

The relationship between insulin and NCC has been previously reported. Studies using distal convoluted tubule (DCT) cell lines have shown that stimulation with a high concentration of insulin enhances NCC activity [15, 20]. In the present study, using an ex vivo kidney slice system, we investigated the effects of insulin on NCC regulation under high potassium conditions. Although the insulin concentration used was at supra-physiological levels, our results suggest that insulin may attenuate the suppression of NCC activity induced by high potassium levels, indicating that insulin may influence NCC responsiveness to changes in electrolyte balance in vivo. In contrast, insulin treatment alone did not alter NCC activity in the present study. This discrepancy may be explained by a difference in the stimulation period. Previous studies reported increases in the pNCC/NCC ratio after 40–60 min of insulin stimulation, whereas the stimulation period in the present study was limited to 30 min. Thus, insulin-induced changes in NCC activity may not have been detectable under the present experimental conditions.

Overall, our findings suggest that DIO may attenuate NCC responsiveness to NaCl loading. Consistent with this, urinary sodium excretion during NaCl loading was reduced in obese mice, suggesting that renal sodium reabsorption was not adequately suppressed. Nevertheless, additional increases in blood pressure in response to NaCl loading were not observed in the DIO groups. It is generally accepted that salt-sensitive hypertension is primarily caused by an increase in circulating blood volume, and that dysregulation of the pressure-natriuresis relationship is a principal underlying mechanism. However, recent studies have suggested that, in addition to increased circulating blood volume, inappropriate vasoconstriction and interstitial sodium retention also contributes to the development of salt-sensitive hypertension [30–32]. A previous study reported that endothelium-dependent relaxation (EDR), a marker of vasodilation, did not differ between mice fed a high-fat diet and those fed a high-fat/high-salt diet after 12 weeks [33]. Therefore, vascular function may have been preserved in the HF+Na group in the present study, potentially explaining the absence of a significant increase in blood pressure in response to NaCl loading.

Regarding the mechanism by which DIO affects NCC responsiveness to NaCl loading, we focused on the WNK-SPAK pathway, a major signaling pathway regulating NCC activity. However, in the present study, no significant changes in SPAK phosphorylation were observed in either the in vivo or ex vivo experiments. Consistent with our findings, previous studies showed that although NCC phosphorylation was suppressed in the kidneys of potassium-loaded mice, corresponding changes in SPAK phosphorylation were not detected by Western blotting of whole-kidney homogenates [34, 35]. Mukherjee et al. [35] suggested that this may be because SPAK is widely expressed throughout the nephron, whereas its phosphorylation occur only in specific nephron segments.

NCC phosphorylation in our ex vivo experiments may have been regulated by insulin through signaling mechanisms other than SPAK. Previous studies have demonstrated that inhibition of PI3K with a specific inhibitor attenuated insulin-induced NCC phosphorylation in cultured DCT cells [15, 20]. Consistent with these findings, the supraphysiological concentration of insulin used in our ex vivo experiments may have influenced NCC phosphorylation through the PI3K/Akt pathway. In addition, several kinases and phosphatases involved in NCC regulation have recently been identified, including Ca^2+^/calmodulin-dependent protein kinase (CaMK) [36, 37] and the Ca²⁺-dependent phosphatase calcineurin (CaN) [34, 38, 39]. Notably, lipocalin 2, which is secreted by several cell types, has been reported to induce NCC phosphorylation via CaMK in a paracrine manner [37], suggesting that intercellular signaling contributes to NCC activation. Accordingly, the ex vivo kidney slice system may preserve intercellular regulatory pathways that are not fully reproduced in conventional in vitro systems. Another possibility is that, because CaN plays a critical role in potassium-induced suppression of NCC phosphorylation [34], the high concentration of insulin used in our ex vivo experiments may attenuate CaN-mediated dephosphorylation rather than enhance SPAK-dependent phosphorylation.

This study has several limitations. In in vivo experiments, the relationship between NCC activation and renal sodium retention was not directly evaluated. Although renin gene expression and serum aldosterone were similarly suppressed in both the high-salt and high-salt-DIO groups, circulating blood volume and vascular resistance were not assessed. In addition, blood pressure and urinary sodium excretion were assessed at only a single time point, limiting the evaluation of their temporal changes and causal relationships. Finally, whether insulin directly regulates NCC activity in vivo was not experimentally confirmed. In the ex vivo kidney slice experiments, the insulin concentration used was considerably higher than physiological circulating levels. Although we used 500 nM insulin based on a previous study [40], it is several hundred times higher than the serum levels observed in DIO mice (2.6–3.2 ng/mL, approximately 0.7–0.9 nM) and therefore may not fully reflect in vivo conditions. Accordingly, the present findings should be interpreted with caution, and future studies using physiologically relevant insulin concentrations are needed.

In conclusion, the present study suggests that DIO may attenuate the normal suppression of NCC activity induced by NaCl loading and that obesity-associated hyperinsulinemia may contribute to this dysregulation. These findings provide insight into altered renal sodium handling in obesity, which may contribute to abnormalities in blood pressure regulation.

## Supplementary information

Supplementary Table 1

Supplementary Table 2
