# Supplementary material for: Diet-induced obesity impairs salt-dependent downregulation of NCC activity
Source: Hypertens Res. 2026 Sep 11;49(11):3114–23. doi: 10.1038/s41440-026-02798-z (PMC13736654; doi:10.1038/s41440-026-02798-z)
Supplement: Supplementary file 1 [file 41440_2026_2798_MOESM1_ESM.docx]

| Nutrients (g) | Standard diet | High-fat diet |
| --- | --- | --- |
| Casein | 14 | 25.6 |
| L-cystine | 0.18 | 0.36 |
| Corn oil | 4 | 2 |
| Lard | 0 | 33 |
| Corn starch | 49.57 | 0 |
| α-corn starch | 12.5 | 22 |
| Sucrose | 10 | 5.5 |
| Cellulose | 5 | 6.61 |
| Vitamin mix | 1 | 1 |
| Choline bitartrate | 0.25 | 0.25 |
| Mineral mix | 3.5 | 3.5 |
| Calcium carbonate | 0 | 0.18 |
| NaCl | 0 | 0 |
| Total (g) | 100 | 100 |
| Energy [kcal/100 g] | 381 | 530 |
| Protein [kcal/100g (% of energy)] | 56.7 (15) | 104 (20) |
| Fat [kcal/100 g (% of energy)] | 36 (9) | 316 (60) |
| Carbohydrate [kcal/100g (% of energy)] | 288.3 (76) | 110 (20) |

Supplementary Table 1. Composition of each diet
