# Supplementary material for: Diet-induced obesity impairs salt-dependent downregulation of NCC activity
Source: Hypertens Res. 2026 Sep 11;49(11):3114–23. doi: 10.1038/s41440-026-02798-z (PMC13736654; doi:10.1038/s41440-026-02798-z)
Supplement: Supplementary file 2 [file 41440_2026_2798_MOESM2_ESM.docx]

Supplementary Table 2. Primer sequences for the quantitative PCR

|  | Forward | Reverse |
| --- | --- | --- |
| *Gapdh* | ATGCCTGCTTCACCACCTTC | GTCGTGGATCTGACGTGCC |
| *Ncc* | CATCTTCTTCCCTTCGGCCA | GTTCCCTTGGGGATGGCTAC |
| *Renin* | TGAAGAAGGCTGTGCGGTAGT | TCCCAGGTCAAAGGAAATGTC |
